# Antimicrobial solid media for screening non‐sterile *Arabidopsis thaliana* seeds

**DOI:** 10.1111/ppl.13079

**Published:** 2020-03-14

**Authors:** James B.Y.H. Behrendorff, Guillem Borràs‐Gas, Mathias Pribil

**Affiliations:** ^1^ Copenhagen Plant Science Centre, Department of Plant and Environmental Sciences University of Copenhagen Frederiksberg Denmark

## Abstract

Stable genetic transformation of plants is a low‐efficiency process, and identification of positive transformants usually relies on screening for expression of a co‐transformed marker gene. Often this involves germinating seeds on solid media containing a selection reagent. Germination on solid media requires surface sterilization of seeds and careful aseptic technique to prevent microbial contamination, but surface sterilization techniques are time consuming and can cause seed mortality if not performed carefully. We developed an antimicrobial cocktail that can be added to solid media to inhibit bacterial and fungal growth without impairing germination, allowing us to bypass the surface sterilization step. Adding a combination of terbinafine (1 μM) and timentin (200 mg l^−1^) to Murashige and Skoog agar delayed the onset of observable microbial growth and did not affect germination of non‐sterile seeds from 10 different wild‐type and mutant *Arabidopsis thaliana* accessions. We named this antimicrobial solid medium “MSTT agar”. Seedlings sown in non‐sterile conditions could be maintained on MSTT agar for up to a week without observable contamination. This medium was compatible with rapid screening methods for hygromycin B, phosphinothricin (BASTA) and nourseothricin resistance genes, meaning that positive transformants can be identified from non‐sterile seeds in as little as 4 days after stratification, and transferred to soil before the onset of visible microbial contamination. By using MSTT agar we were able to select genetic transformants on solid media without seed surface sterilization, eliminating a tedious and time‐consuming step.

**Abbreviations**
BASTA: phosphinothricin or glufosinate
mEGFP: monomeric enhanced green fluorescent protein
MS: Murashige and Skoog medium
MSTT: Murashige and Skoog medium with terbinafine and timentin
MSTT+suc: MSTT with sucrose
PDS3: phytoene desaturase 3

## Introduction

Driven by cheap and reliable methods of DNA assembly, the synthetic biology revolution has made it possible for molecular biologists to design and build dozens of new plasmids in as little as 1 or 2 weeks even without automation equipment. Plant science has not fully exploited these advances in molecular cloning to the same extent as other disciplines, partly because of experimental throughput limitations unique to plants.

Agrobacterium‐mediated genetic transformation is one of the most versatile and accessible methods for modifying the genome of *Arabidopsis thaliana* (Bechtold and Pelletier 1998, Zhang et al. 2006), but this approach produces only a small minority of seeds in the T_1_ generation that carry the transgene of interest. Transformation efficiencies between 0.57 and 2.57% have been reported with optimized variations of the classic floral dipping method (Chung et al. 2000, Martinez‐Trujillo et al. 2004, Zhang et al. 2006). Identifying this minority of positive transformants usually relies on selection or screening for a co‐transformed marker gene.

The most common selection approaches involve germinating seeds on an agar‐based solid nutrient medium that contains a chemical reagent to select seedlings that express the corresponding marker gene. Popular selectable markers confer resistance to phosphinothricin (BASTA, also known as glufosinate), kanamycin, hygromycin B, or nourseothricin (also known as streptothricin) (Jelenska et al. 2000, Harrison et al. 2006). Germination on solid media requires that seeds are surface sterilized to prevent overgrowth by microbial contaminants during the selection process.

At the time when these screening methods were established, molecular cloning was a bottleneck in the experimental workflow of transgenic plant preparation and typically few transgenic lines were prepared simultaneously. This is no longer the case, yet the same screening methods are still widely used. Screening for successful stable transfection events now represents a significant bottleneck, especially when an experiment involves several different genetic designs.

The seed sterilization step in particular has disadvantages that become more pronounced when screening increasing numbers of transformant lines. Liquid sterilization in hypochlorite bleach has a low seed mortality rate but is tedious, requiring several washing steps that become time consuming when preparing large quantities of seeds (Lindsey et al. 2017). Chlorine gas is suitable for sterilizing seeds from multiple lines simultaneously, but gas sterilization still requires up to 4 h of waiting time and can have a relatively high mortality rate even when the gas concentration is carefully controlled (Lindsey et al. 2017). Mortality caused by the sterilization process could result in the loss of rare transformants or a reduction in the diversity of mutant libraries. Furthermore, surface sterilization does not necessarily eliminate microbial spores that can be trapped inside the seed coat during embryogenesis (Andargie and Li 2016).

We aimed to develop a method that would allow us to avoid surface sterilization of seeds altogether. Our approach was to identify a combination of antifungal and antibacterial compounds that inhibit microbial growth but do not impair *Arabidopsis* germination and growth. The antimicrobial medium presented here (MSTT agar) substantially delays microbial contamination. When combined with established rapid selection methods (Harrison et al. 2006), we were able to identify positive transformants from non‐sterile seeds in less than 1 week and transfer them to soil for growth and propagation prior to the onset of observable microbial contamination.

## Materials and methods

### Chemicals

Murashige and Skoog medium including vitamins (MS medium) (Murashige and Skoog 1962) (Cat. no. M0222), hygromycin B (Cat. no. H0192) and timentin (ticarcillin 2NA and clavulanate K 15:1 mixture, Cat. no. T0190) were purchased from Duchefa Biochemie. Nourseothricin was purchased from Jena Bioscience (Cat. no. AB‐102L). BASTA was purchased from Bayer Cropscience (Product no. 84442615). Terbinafine (Cat. no. T8826) was purchased from Merck. All other chemicals were the highest quality locally available. Stock solutions were prepared as follows: timentin, 200 mg ml^−1^ in water; nourseothricin, 50 mg ml^−1^ in water; terbinafine, 1 mM in dimethylsulfoxide (DMSO); carbenicillin, 50 mg ml^−1^ in water; BASTA, 50 mM in water. Hygromycin B was used directly from the liquid stock provided by the manufacturer (400 mg ml^−1^ in water).

### Plant accessions

*Arabidopsis thaliana* mutants (*curt1abcd* (Armbruster et al. 2013), *atpC1* (Dal Bosco et al. 2004), *hcf136* (Meurer et al. 1998), *pam68* (Armbruster et al. 2010), *psal* (Lunde et al. 2000), and *npq4* (Li et al. 2000) and wild‐type ecotypes (Columbia (Col‐0), Landsberg *erecta* (Ler‐0), Wassilewskija (Ws‐0), and Nossen (No‐0)) were a gift from Prof. Dario Leister, Ludwig Maximilians Universität, Germany. *Nicotiana tabacum* (cv. Petit Havana) seeds were a gift from Dr. Lars Scharff (University of Copenhagen, Denmark).

### Antimicrobial compound screening

MS agar (0.5×) was prepared by dissolving Murashige and Skoog medium in water and adjusting the pH to 5.7 with 1 M potassium hydroxide. Agar was added to a final concentration of 1% (w/v) and sterilized by autoclaving. Sucrose was added from a filter‐sterilized stock solution (40% w/v in water). Antimicrobial reagents were added from the stock solutions described above (in see Chemicals), and all agar plates were poured in non‐sterile conditions on a standard laboratory bench. In all screening experiments, non‐sterile seeds were sown directly onto solidified agar without any pre‐treatment. Throughout this study, seeds were stratified by wrapping the agar plates in aluminum foil and storing at 4°C for 68 h. After stratification, agar plates were maintained under long day conditions (16 h light at 100 μmol m^−2^ s^−1^, 8 h darkness, 22°C, 60% humidity) in a Fitotron SGC 120 growth chamber (Weisstechnik, Germany).

During the development phase of this study we compared the effects of different antimicrobial reagents on germination of 10 different *A. thaliana* lines and *N. tabacum*. Therefore, for each condition tested there were n = 11 paired comparisons between treated and untreated seeds. On each plate, between 23 and 122 seeds were sown.

The final antimicrobial solid medium developed in this study (MSTT agar) contained 0.5× MS medium (pH 5.7), terbinafine (1 μM), timentin (200 mg l^−1^), and agar (10 g l^−1^). MSTT+suc agar also contained sucrose (1%, w/v).

### Plasmids

All cloning strategies were designed with Geneious 10.2.6 (http://www.geneious.com) and performed using the general principles of the Gibson assembly method (Gibson et al. 2009). Cartoon representations of plasmids were generated with Pigeon (Bhatia and Densmore 2013) (http://pigeon.synbiotools.org). Oligonucleotide primers and sources of template DNA are listed online in Appendix S2. *Fluorescent reporter protein constructs*: in a previous study (Behrendorff et al. 2019), a nourseothricin‐selectable plasmid was prepared by replacing the bialaphos resistance (*bar*) gene from plasmid pB2GW7 (Karimi et al. 2002) with the nourseothricin acetyl transferase (*nat*) gene from *Streptomyces noursei*. The resulting plasmid is described as pN_35S. The ccdB counter‐selectable marker was replaced with the coding sequences for either mEGFP or mApple fluorescent proteins, placing fluorescent protein expression under the control of the cauliflower mosaic virus 35S promoter (plasmids pN_35S/mEGFP and pN_35S/mApple are available at www.addgene.org as plasmids #132565 [RRID:Addgene_132 565] and #132566 [RRID:Addgene_132 566], respectively). The pB_35S/mEGFP plasmid was prepared by cloning the mEGFP coding sequence directly into the pB2GW7 backbone, replacing the ccdB counterselectable marker (www.addgene.org, plasmid #135320 [RRID:Addgene_135 320]). We have also made available a hygromycin‐selectable mEGFP expression plasmid, pH_35S/mEGFP (www.addgene.org, plasmid #135321 [RRID:Addgene_135 321]), prepared by cloning the mEGFP coding sequence into the pH2GW7 (Karimi et al. 2002) backbone. Plasmid pN_35S/CTP‐mCitrine was produced in an earlier study (Behrendorff et al. 2019) and encodes an mCitrine fluorescent protein fused in‐frame to the chloroplast transit peptide from RuBisCO small subunit 1A (www.addgene.org, plasmid #117989 [RRID:Addgene_117 989]). *CRISPR constructs*: an mApple fluorescent protein was fused in‐frame to the C‐terminus of a *Streptomyces pyogenes* Cas9 via a GGGGS flexible linker. The Cas9‐mApple coding sequence and a *PDS3* sgRNA under the control of the *A. thaliana* U6 polymerase III promoter (Li et al. 2013) were cloned into the pH2GW7 (Karimi et al. 2002) backbone (hygromycin selection). A previously‐described promoter made by combining two *A. thaliana* egg cell specific promoters (Wang et al. 2015b) was then inserted upstream of the Cas9‐mApple coding sequence to create GS2.1/EC (www.addgene.org, plasmid #132568 [RRID:Addgene_132 568]).

Plasmids were transformed into *Agrobacterium fabrum* strain GV3101 (previously known as *Agrobacterium tumefaciens* GV3101 [Gan and Savka 2018) via electroporation with the following conditions: voltage 2500 V, capacitance 25 μF, resistance 400 Ω, 2 mm cuvette.

### Genetic transformation and screening

*A. thaliana* (Col‐0) was grown under long day conditions (16 h light at 100 μmol m^−2^ s^−1^, 8 h darkness) at 22°C and 60% humidity, and transformed according to a modified floral dip method described previously (Martinez‐Trujillo et al. 2004). Seeds collected from transformed plants were sown on MSTT or MSTT+suc agar with appropriate selection reagents.

Plants resistant to nourseothricin (50 mg l^−1^) or hygromycin B (15 mg l^−1^) were identified by rapid screening for hypocotyl elongation (Harrison et al. 2006). After stratification, plates were shifted to growth chamber conditions and exposed to light for 6 h. Plates were then wrapped in foil to maintain darkness for 2 full days and stored at 22°C. On the fourth day, plates were unwrapped and resistant seedlings with elongated hypocotyls were clearly distinguishable from non‐resistant seedlings. Plants resistant to BASTA (50 μM) were identified by rapid screening for green expanded cotyledons (Harrison et al. 2006). Plates were shifted to growth chamber conditions after stratification and exposed to light for 6 h, and then wrapped in foil to maintain darkness for 3 full days. Following the dark treatment, plates were unwrapped and kept in a growth chamber under long day conditions. Positive transformants could be identified 2 days later (i.e. the fifth day post‐stratification), and differences between resistant and non‐resistant seedlings were more pronounced on the sixth day post‐stratification.

In the case of plants transformed with fluorescent protein expression constructs (pN_35S/mEGFP, pN_35S/CTP‐mCitrine, pN_35S/mApple, or pB_35S/mEGFP), transformation was verified by fluorescence imaging on a Bio‐Rad ChemiDoc XRS+ (Bio‐Rad Laboratories, Inc.). Green and yellow fluorescent signals (from mEGFP and chloroplast‐targeted mCitrine) were captured using blue light epi‐illumination and a 530 nm filter (28 nm bandpass). Red fluorescence from mApple was captured with green light epi illumination and a 605 nm filter (50 nm bandpass). For red fluorescence imaging, it was necessary to image seedlings on the fourth day post‐stratification, prior to greening of cotyledons (i.e. on the same day that the germinating seedlings were removed from dark treatment). It was not possible to identify mApple fluorescence in green leaves due to interference from chlorophyll autofluorescence.

Homozygous *pds3* mutants were identified by their distinct albino phenotype and were confirmed via Sanger sequencing (oligonucleotide primer details included online in Appendix S2).

### PCR from leaf tissue

Diagnostic PCRs, preparative PCRs for Sanger sequencing, and PCRs to prepare *A. thaliana* DNA for cloning were performed using leaf tissue as the source of template DNA. A portion of leaf tissue (approximately 5 mm^2^) was homogenized by grinding in 50 μl of 1× Q5 reaction buffer (New England Biolabs Cat. No. B9027S) in a 1.5 ml microcentrifuge tube with a micropestle. The homogenate was heated to 98°C for 10 min, then cooled on ice. After cooling, leaf debris was separated by centrifugation (30 s, 13000 *g*). The supernatant was used directly as a source of template DNA (1 μl template DNA per PCR).

## Results

### Terbinafine as an antifungal reagent

Terbinafine is an antifungal reagent that inhibits squalene epoxidase, causing a deficiency in the membrane lipid ergosterol (Ryder 1992). Squalene epoxidation is also a key step in the biosynthesis of plant sterols, and squalene epoxidase knockout mutants of *A. thaliana* Col‐0 exhibit increased sensitivity toward terbinafine while wild‐type plants appear phenotypically normal post‐germination (Laranjeira et al. 2015). We sought to test whether low concentrations of terbinafine could be used to inhibit fungal growth without impairing germination of *A. thaliana*.

In a preliminary experiment, non‐sterile seeds from four wild‐type *A. thaliana* ecotypes (Columbia (Col‐0), Landsberg *erecta* (Ler‐0), Wassilewskija (Ws‐0), and Nossen (No‐0)), six photosynthetic gene mutants [*curt1abcd* (Armbruster et al. 2013), *atpC1* (Dal Bosco et al. 2004), *hcf136* (Meurer et al. 1998), *pam68* (Armbruster et al. 2010), *psaL* (Lunde et al. 2000), and *npq4* (Li et al. 2000)], and *Nicotiana tabacum* (cv. Petit Havana) were sown directly onto 0.5× Murashige and Skoog (MS) agar with sucrose (1%, w/v) and terbinafine (added to a final concentration of 1, 0.1, or 0.01 μM). Negative control plates contained DMSO (0.1%, v/v) without terbinafine. While sucrose is not necessary for germination of wild‐type plants, many mutants with impaired photosynthesis benefit from the addition of sucrose during germination. Sucrose also increases the risk of microbial contamination because it is a utilizable carbon source for most fungi and many bacteria. Therefore, we included sucrose in our media to ensure that our antimicrobial medium would be useful in cases where the inclusion of sucrose is necessary.

The onset of germination (defined as the first cotyledons to emerge on each plate) was determined by visual inspection and was scored qualitatively, as was the emergence of observable microbial contamination. Plates were inspected twice per day for 7 days (168 h in total).

Terbinafine did not affect the onset of germination at any of the concentrations tested (up to 1 μM) (online in Fig. S1A). In negative control agar plates that lacked terbinafine, microbial contamination was observed as early as 24 h after being transferred to growth chamber conditions (median time to visible contamination: 64 h) (Fig. S1B). In the presence of terbinafine, there was a general trend toward delayed onset of microbial contamination with increasing terbinafine concentration. At 1 μM terbinafine, all plates were free of microbial contamination after 168 h except for one plate, where a contaminant emerged after 112 h. It was not obvious whether the contaminant was bacterial or fungal, but these results indicate that 1 μM terbinafine decreases the likelihood of fungal contamination and/or delays fungal growth.

### Combining terbinafine with antibacterial β‐lactam antibiotics

β‐lactam antibiotics were tested as the antibacterial reagent because they inhibit peptidoglycan biosynthesis in prokaryotes, whereas most other classes of prokaryote‐targeting antibiotics also interfere with plastid (Ellis 1969, Mulo et al. 2003) and mitochondrial protein synthesis (Wang et al. 2015a). We examined the effects of carbenicillin and timentin, which are both commonly used for eliminating Agrobacteria from plant tissue culture (Lin et al. 1995, Zhang et al. 2006, Yan et al. 2015). Timentin is a mixture containing a β‐lactam antibiotic (ticarcillin) and a β‐lactamase inhibitor (clavulanic acid).

Timentin (200 mg l^−1^) or carbenicillin (500 mg l^−1^) was added to 0.5× MS agar that contained sucrose (1%, w/v) and terbinafine (1 μM). Non‐sterile seeds were sown directly onto agar plates and stratified as described above, and then transferred to growth chambers and monitored by visual inspection. Germination was quantified by recording the number of germinated seedlings twice per day for the first 4 days, and once per day thereafter. Microbial contamination was recorded qualitatively.

**Figure 1:**
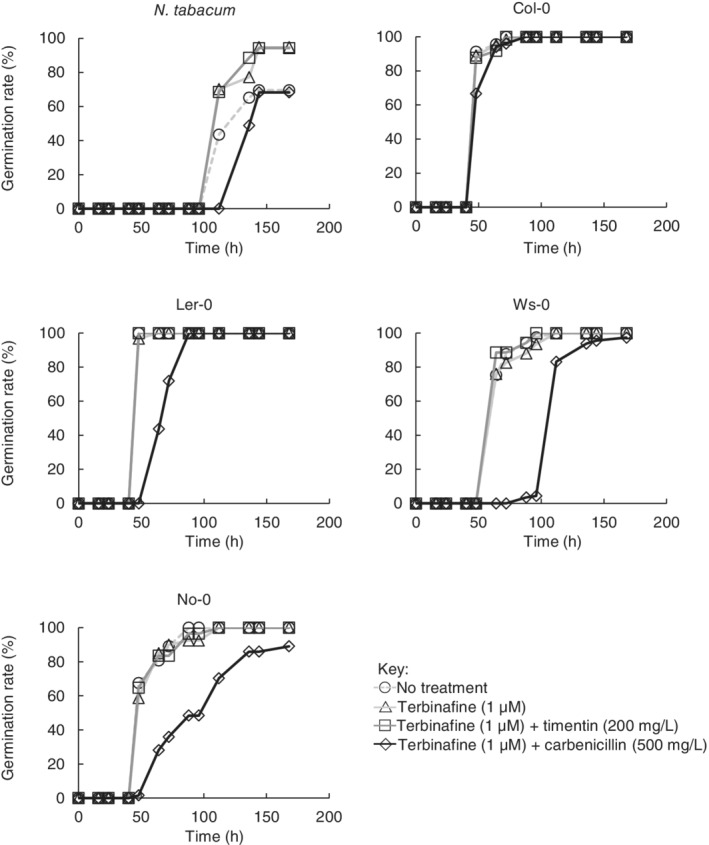
Germination of wild‐type seeds in the presence of terbinafine and β‐lactam antibiotics. Non‐sterile seeds for *Nicotiana tabacum* (cv. Petit Havana) and *Arabidopsis thaliana* ecotypes Columbia (Col‐0), Landsberg *erecta* (Ler‐0), Wassilewskija (Ws‐0), and Nossen (No‐0) were sown on 0.5× MS agar with added sucrose (1%, w/v) and different combinations of terbinafine and timentin or carbenicillin (indicated). Germination was monitored by visual inspection and the number of germinated seeds was recorded as a percentage of the total seeds sown on that agar plate. N > 40 seeds per plate, except for *N. tabacum* (n > 16 seeds per condition).

**Figure 2:**
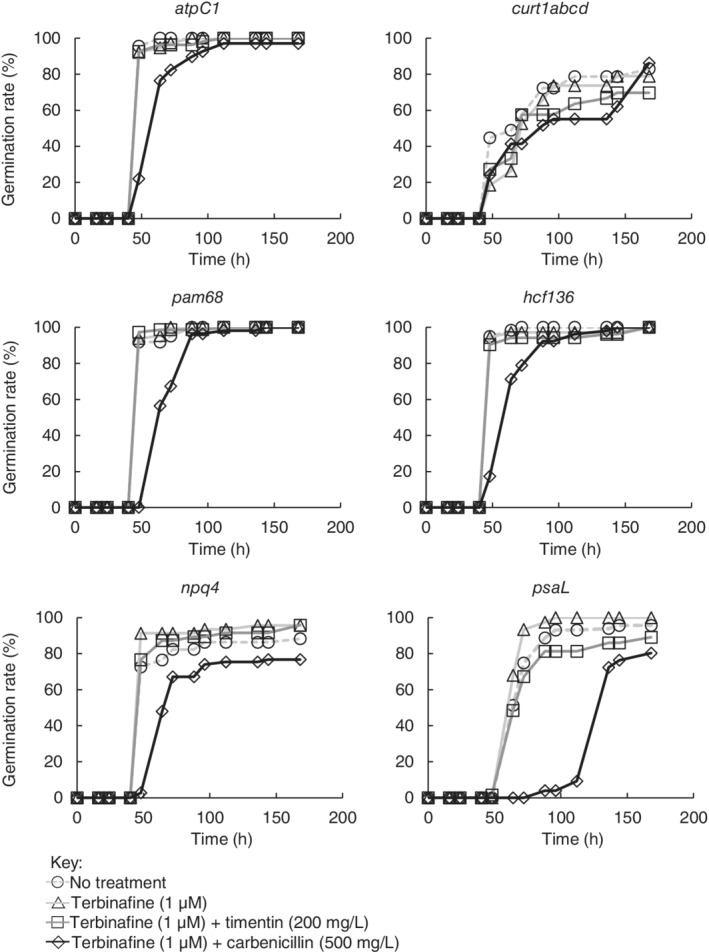
Germination of *Arabidopsis thaliana* photosynthetic mutants in the presence of terbinafine and β‐lactam antibiotics. Non‐sterile seeds for six *A. thaliana* photosynthetic mutants (*atpC1*, *curt1abcd*, *pam86*, *hcf136*, *npq4* and *psaL*) were sown on 0.5× MS agar with added sucrose (1%, w/v) and different combinations of terbinafine and timentin or carbenicillin (indicated). Germination was monitored by visual inspection and the number of germinated seeds was recorded as a percentage of the total seeds sown on that agar plate (n > 25 seeds per condition).

**Figure 3:**
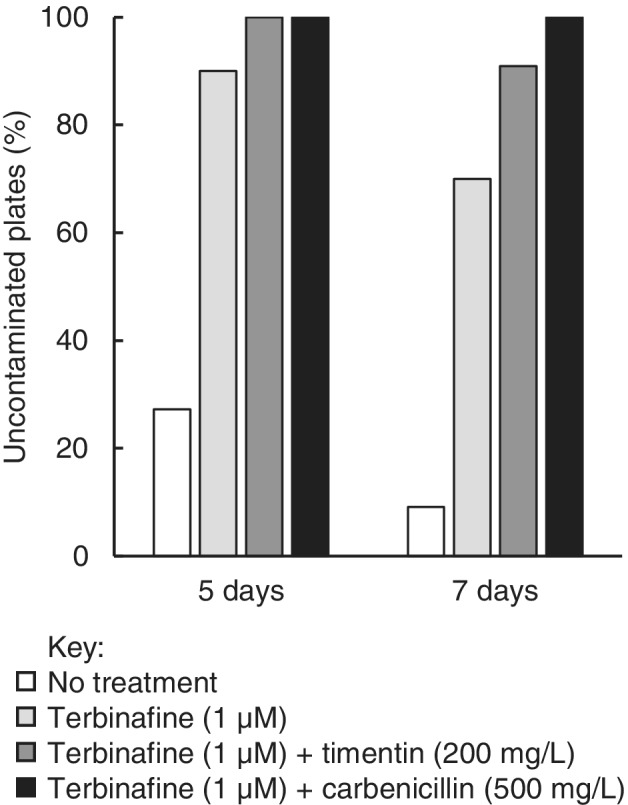
Onset of microbial contamination in the presence of terbinafine and β‐lactam antibiotics. Non‐sterile seeds were sown on 0.5× MS agar with added sucrose (1%, w/v) and different combinations of terbinafine and timentin or carbenicillin (indicated). The proportion of agar plates that remained uncontaminated after 5 and 7 days was recorded (n = 11 agar plates per condition).

The combination of terbinafine (1 μM) and timentin (200 mg l^−1^) did not inhibit germination of wild‐type *A. thaliana* ecotypes or *N. tabacum* compared with untreated seeds (sown on 0.5× MS agar + sucrose without antimicrobial additives) (Fig. 1). Photosynthetic mutant *A. thaliana* lines were also unaffected (Fig. 2) except in the case of the *psaL* mutant, where the inclusion of timentin may have impaired germination in 7% of seeds (at 168 h: 96% of untreated seeds had germinated vs 89% of seeds sown on timentin plus terbinafine). Carbenicillin (500 mg l^−1^) inhibited normal root development in all lines examined and delayed germination in all cases except for the *curt1abcd* quadruple mutant, which exhibits slower germination than the wild‐type ecotypes examined here and naturally produces a lower proportion of viable seeds (Pribil et al. 2018). The combination of terbinafine (1 μM) and timentin (200 mg l^−1^) prevented microbial contamination for 5 days in 100% of cases, and for 7 days in 90% of cases (Fig. 3). Henceforth we describe 0.5× MS agar containing this combination of terbinafine and timentin as MSTT agar, or MSTT+suc agar when the medium also contains sucrose (1% w/v).

Germination of the four wild‐type *A. thaliana* ecotypes and *N. tabacum* was also examined on MSTT agar without sucrose. Non‐sterile seeds were sown on 0.5× MS agar or MSTT agar, and germination was unimpeded in all cases (Fig. S2). Contamination emerged on negative control plates (0.5× MS agar) after only 48 h, whereas MSTT agar plates remained free of observable contamination for 1 week (Fig. S3). Microbial contamination emerged on all MSTT agar plates after 184 h. After germination, plants transferred to soil from MSTT and MSTT+suc agar developed into phenotypically normal adult plants, indistinguishable from controls sown directly on soil (Fig. S4).

*N. tabacum* germination is slightly accelerated by the presence of terbinafine (1 μM) and slightly delayed by the presence of sucrose (Fig. 1, Fig. S2, and additional experiments not shown here). We observe these effects consistently when germinating *N. tabacum* but have been unable to find a satisfactory explanation in the scientific literature.

### Non‐sterile screening for *Arabidopsis* transformants on selective agar

#### *Screening for nourseothricin resistance and fluorescent protein expression*

**Figure 4:**
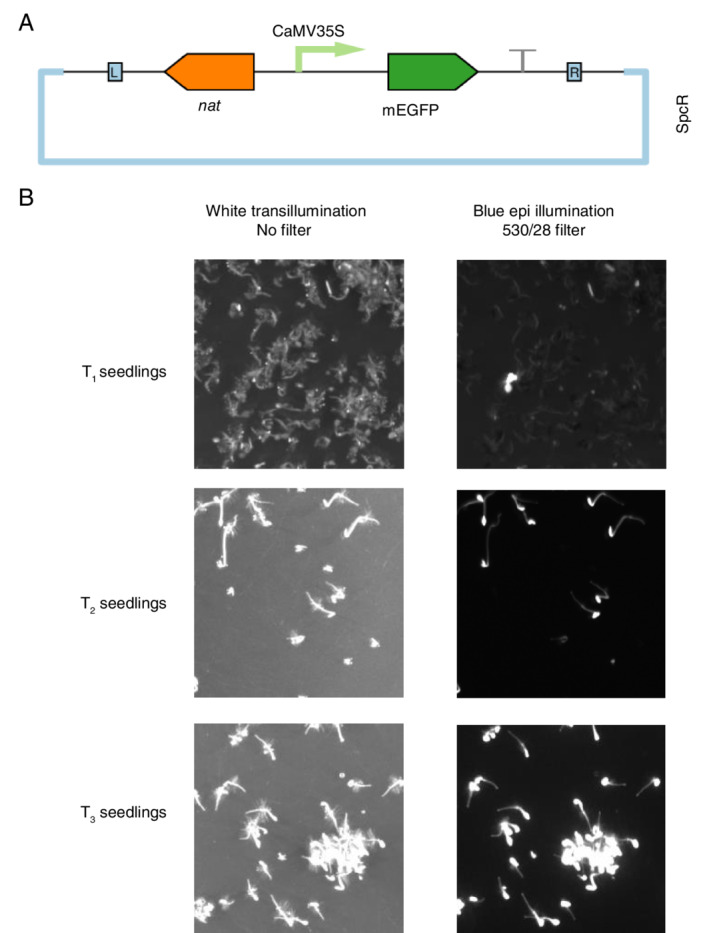
Screening for nourseothricin‐resistant transformants with non‐sterile seeds. *Arabidopsis thaliana* (Col‐0) was transfected with a green fluorescent protein expression construct and the resulting seeds were screened for transgene integration in non‐sterile conditions on MSTT agar with added nourseothricin (50 mg ml^−1^). (A) Schematic map of the plasmid used for Agrobacterium‐mediated transfection. The T‐DNA region is flanked by L and R, indicating the left and right border sequences. Monomeric enhanced green fluorescent protein (mEGFP) expression is regulated by the cauliflower mosaic virus 35S promoter (CaMV35S). Nourseothricin acetyl transferase (*nat*) is the selectable marker for plant transformation, and SpcR indicates that the plasmid backbone confers resistance to spectinomycin in bacteria. (B) The screening procedure identified positive transformant T_1_ plants and was repeated to identify homozygous plants in the T_3_ generation. Transgene integration and expression was confirmed by screening for hypocotyl elongation (indicating *nat* expression) and mEGFP fluorescence (imaged by illumination with a blue light source and a 530 nm filter with a 28 nm bandpass).

Having established that MSTT agar delays microbial contamination without impairing germination, we examined whether this medium would allow us to screen *A. thaliana* genetic transformants without seed sterilization. As a test case, we transfected *A. thaliana* (Col‐0) with a green fluorescent protein expression construct, pN_35S/mEGFP: a monomeric enhanced green fluorescent protein (mEGFP) under the control of the cauliflower mosaic virus 35S promoter, with a nourseothricin acetyl transferase (*nat*) selectable marker (Fig. 4A). Non‐sterile seeds collected from the T_0_ plant were sown directly onto MSTT agar with added nourseothricin (50 mg l^−1^). Seeds were stratified directly on the agar plates and then screened using the rapid hypocotyl elongation method that was previously developed for identifying hygromycin B resistance (Harrison et al. 2006). Briefly, this method involves exposing stratified seeds to light for 6 h to break dormancy, and then keeping the germinating seedlings in darkness at 22°C to promote hypocotyl elongation. Seedlings that express the resistance marker gene grow elongated hypocotyls, whereas non‐transformed plants do not (Harrison et al. 2006).

Plates were uncovered after 2 full days of dark treatment (i.e. on the fourth day post‐stratification). Individual seedlings with extended hypocotyls were clearly identifiable amongst the majority of seedlings that did not have extended hypocotyls, and fluorescence imaging revealed mEGFP expression (Fig. 4B) in the same seedlings that had extended hypocotyls. No microbial growth was observed and several positive transformants were transferred to soil for propagation. This demonstrated that MSTT agar can be used to limit microbial growth during nourseothricin‐based screening, and that nourseothricin is compatible with the hypocotyl elongation rapid screening method. Homozygous transformant lines were identified by repeating the screening procedure with seeds collected from T_1_ and T_2_ plants (Fig. 4B).

We used the same method to identify plants transformed to overexpress the mApple red fluorescent protein (transformed with pN_35S/mApple) and a nuclear‐encoded chloroplast‐targeted mCitrine yellow fluorescent protein (pN_35S/CTP‐mCitrine) (Fig. S5).

#### *Screening for BASTA resistance and fluorescent protein expression*

**Figure 5:**
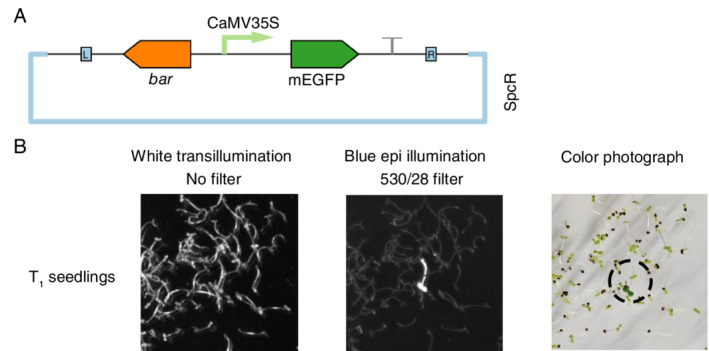
Screening for BASTA‐resistant transformants with non‐sterile seeds. *Arabidopsis thaliana* (Col‐0) was transfected with a green fluorescent protein expression construct and the resulting seeds were screened for transgene integration in non‐sterile conditions on MSTT agar with added BASTA (50 μM). (A) Schematic map of the plasmid used for agrobacterium‐mediated transfection. The T‐DNA region is flanked by L and R, indicating the left and right border sequences. Monomeric enhanced green fluorescent protein (mEGFP) expression is regulated by the cauliflower mosaic virus 35S promoter (CaMV35S). Phosphinothricin *N*‐acetyl transferase (*bar*) is the selectable marker for plant transformation, and SpcR indicates that the plasmid backbone confers resistance to spectinomycin in bacteria. (B) The screening procedure identified positive transformant T_1_ plants. Transgene integration and expression was confirmed by screening for cotyledon expansion and greening (indicating *bar* expression) and mEGFP fluorescence (imaged by illumination with a blue light source and a 530 nm filter with a 28 nm bandpass). The positive transformant, distinguishable by its larger, dark green cotyledons, is circled with a dashed line in the color photograph panel.

We also tested whether non‐sterile germination on MSTT agar could be coupled with rapid phosphinothricin (BASTA)‐based screening. *A. thaliana* (Col‐0) plants were transfected with the pB_35S/mEGFP expression construct (identical to pN_35S/mEGFP except that it has a phosphinothricin N‐acetyltransferase (*bar*) selectable marker in place of the nourseothricin acetyl transferase gene) (Fig. 5A). Seeds from the T_0_ plant were sown directly onto MSTT agar with added BASTA (50 μM) and stratified as described above. BASTA‐resistant seedlings were identified using the previously published rapid screening method (Harrison et al. 2006) with minor modifications. Briefly, stratified seeds were exposed to light for 6 h to break dormancy, and then kept in darkness at 22°C for 3 days before transferring to long day growth chamber conditions (see Methods for details). After 2 days in growth chamber conditions, transformed seedlings were clearly identifiable by their dark green expanded cotyledons while non‐transformed seedlings exhibited pale unexpanded cotyledons. These phenotypic differences became more pronounced after 3 days in long day conditions. Fluorescence imaging confirmed mEGFP expression in seedlings with green expanded cotyledons (Fig. 5B). No microbial growth was observed during the screening period and multiple positive transformants were identified.

#### *Screening for hygromycin B resistance and identifying mutants produced with CRISPR‐Cas9*

**Figure 6:**
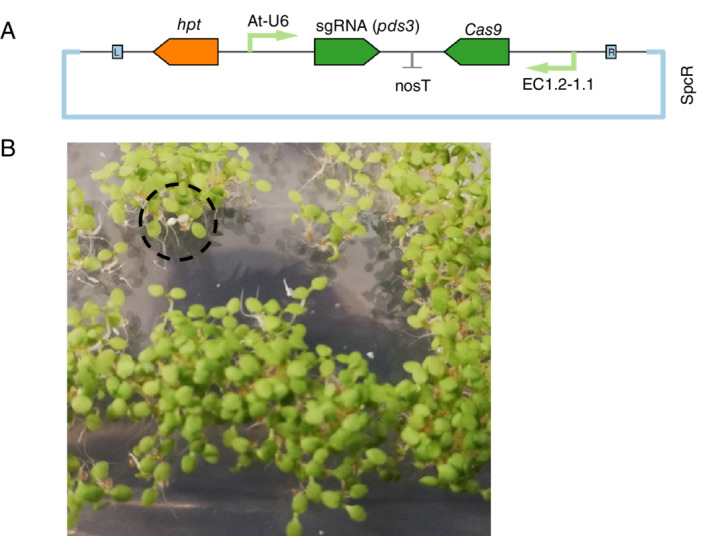
Screening for a CRISPR‐Cas9‐mediated *pds3* mutant phenotype with non‐sterile seeds. *Arabidopsis thaliana* (Col‐0) was transfected with a CRISPR‐Cas9 plasmid targeting mutation of the phytoene desaturase, *PDS3*. Homozygous *pds3* mutants were obtained by screening seeds in non‐sterile conditions on MSTT+suc agar with added hygromycin B (15 mg l^−1^). (A) Schematic map of the plasmid used for agrobacterium‐mediated transfection. The T‐DNA region is flanked by L and R, indicating the left and right border sequences. A single guide RNA (sgRNA) targeting *PDS3* is regulated by the *A. thaliana* U6 polymerase III promoter (At‐U6). The Cas9 gene is regulated by an egg cell‐specific promoter (EC1.2–1.1). The hygromycin phosphotransferase (*hpt*) is the selectable marker for plant transformation, and SpcR indicates that the plasmid backbone confers resistance to spectinomycin. (B) Non‐sterile seeds were screened on MSTT+suc agar with hygromycin B. Homozygous *pds3* knockout mutants were identifiable in the T_2_ generation by their characteristic albino phenotype. An example *pds3* mutant on day five post‐stratification is indicated inside the dashed circle.

In a third test case, we transfected *A. thaliana* (Col‐0) with a construct for CRISPR‐Cas9‐mediated functional knockout of the phytoene desaturase (*PDS3*) gene, which causes an albino phenotype (Qin et al. 2007). Our plasmid (GS2.1/EC) combined a previously published *PDS3*‐targeting sgRNA (Li et al. 2013) with a Cas9 gene under the control of a previously published egg cell‐specific promoter (Wang et al. 2015b). The T‐DNA region also carried a hygromycin phosphotransferase gene conferring resistance to hygromycin B (Fig. 6A).

Non‐sterile seeds collected from T_0_ plants were sown on MSTT+suc agar with hygromycin B (15 mg l^−1^). Positive transformants were identified on the basis of hypocotyl elongation on the fourth day post‐stratification. T‐DNA integration was confirmed by PCR analysis of leaf tissue (Fig. S6), but all positive transformants in the T_1_ generation had green cotyledons indicating that any post‐transfection CRISPR‐Cas9 activity during seed development had not resulted in homozygous *pds3* mutants.

Non‐sterile seeds collected from one T_1_ plant were sown on MSTT+suc agar containing hygromycin B (15 mg l^−1^). Approximately 1500 seeds were sown on a single 12 × 12 cm agar plate. Approximately 75% of seedlings were resistant to hygromycin B, and on the fifth day post‐stratification (i.e. after cotyledon greening) three albino *pds3* mutants were identified (Fig. 6B). The three albino seedlings were confirmed as independent *pds3* mutants using Sanger sequencing (Fig. S7).

## Discussion

When producing new *A. thaliana* transgenic lines, experimental throughput is partly limited by the transformant screening process. In particular, the seed sterilization step is time consuming and can cause seed mortality (Lindsey et al. 2017). Alternative screening methods that negate the need for seed sterilization have been developed, each with advantages and disadvantages.

Conventional selection for BASTA resistance (conferred by *bar*, the phosphinothricin N‐acetlytransferase gene) (D'Halluin et al. 1992) involves spraying the aerial parts of germinated seedlings and can be performed in non‐sterile conditions with seeds sown directly on soil at relatively high densities. The disadvantage of this approach is the time required to identify positive transformants: typically at least three spray applications are spread across 3 weeks (Leclere and Bartel 2001). Additionally, the use of BASTA is restricted in some countries due to concerns surrounding neurotoxicity linked to BASTA ingestion (Hack et al. 1994, Watanabe and Sano 1998, Mao et al. 2011).

A more modern approach to avoiding seed sterilization is to use a fluorescent protein with seed‐specific expression as the marker gene (Stuitje et al. 2003, Shimada et al. 2010). Accumulation of the fluorescent protein in transformed seeds can be observed visually with a suitable light source and filter combination (a fluorescence microscope is typically used). Visual screening for a co‐expressed fluorescent protein avoids the need for sterilization, and only seeds with active transgene expression are sown on soil. This is an excellent approach for avoiding seed mortality and reducing the number of plants that need to be grown in a screening campaign, but this approach is still best suited to scenarios involving relatively few transgenic lines due to the labour involved in screening seeds under a fluorescence microscope.

The method we present here provides another pragmatic option for avoiding seed sterilization. When paired with rapid screening methods, seeds sown on MSTT or MSTT+suc agar could be screened for resistance to nourseothricin, hygromycin B or BASTA in as few as 4–5 days after stratification. This approach allowed us to curate homozygous T_3_ transgenic lines and identify CRISPR‐Cas9‐mediated *pds3* knockout mutants without the need for seed sterilization at any stage. It is possible that the transgene selection reagents also contribute to the antimicrobial effect when used in combination with terbinafine and timentin, but nourseothricin and hygromycin B were not sufficient to prevent contamination when used alone (data not shown).

The MSTT formulation that we developed can be used to delay the onset of microbial growth or decrease the likelihood of contaminants becoming established, but it cannot be used to maintain sterile conditions outright. Therefore, we can only recommend its use with species that germinate quickly (i.e. in less than a week). Additionally, all seeds used in this study were produced in growth chamber conditions; our method may not be suitable for use with field‐grown seeds that could be expected to have a greater microbial burden.

Although MSTT and MSTT+suc agar did not negatively affect germination of any of the seeds examined in this study, we only validated these media for use in screening laboratory‐grown seeds for transgene insertion. It is unknown whether exposure to sub‐inhibitory concentrations of terbinafine and timentin may trigger any responses in *Arabidopsis* that would make these media unsuitable for physiological studies.

When developing the MSTT formulation, we identified terbinafine as a candidate antifungal reagent from an earlier study that investigated squalene metabolism in Arabidopsis (Laranjeira et al. 2015). We also tested miconazole (20 mg l^−1^) on the basis that it had been used to prevent fungal overgrowth in explant tissue culture of a number of crop species (Tynan et al. 1993), but miconazole was 100% lethal to all of our Arabidopsis lines at this concentration (data not shown). It is possible that miconazole would be useful at lower concentrations but we did not pursue this further given our immediate success with terbinafine.

While the inhibitory effect of terbinafine on squalene epoxidase in plants has been characterized, the effects of β‐lactam antibiotics on plants are not fully understood. Peptidoglycan biosynthesis is retained in moss chloroplasts but is absent from vascular plants (Hirano et al. 2016), and it is generally assumed that β‐lactam antibiotics do not affect the chloroplasts of higher plants (Reski 2009). Carbenicillin (500 mg l^−1^) has been described in the literature as beneficial for eliminating β‐lactam‐sensitive *Agrobacterium* strains from transfected *Arabidopsis* and tobacco tissue culture (Lin et al. 1995), and carbencillin concentrations between 100 and 500 mg l^−1^ have also been recommended for use in solid media when screening T_1_ transgenic *Arabidopsis* seeds after floral dip transformation (Zhang et al. 2006, Cold Spring Harbor Protocols 2010, Yan et al. 2015). We initially planned to use a high concentration of carbenicillin (500 mg l^−1^) on the basis that β‐lactamase enzymes are secreted by many environmental bacteria and some commonly used laboratory strains of Agrobacteria (Ogawa and Mii 2004). However, carbenicillin and penicillin were recently reported to impair root elongation in *A. thaliana* at concentrations between 100 and 1000 mg l^−1^L (Gudiño et al. 2018). As an alternative to carbenicillin, we considered timentin on the basis that a lower concentration of timentin should provide a similar protective effect due to the presence of a β‐lactamase inhibitor (clavulanic acid) in the timentin formulation. We observed that carbenicillin (500 mg l^−1^) did not cause seed mortality but delayed germination and prevented root elongation, whereas timentin (200 mg l^−1^) had no observable effect on *Arabidopsis* germination and growth.

In conclusion, timentin and terbinafine added to 0.5× MS agar delay the onset of microbial contamination and do not inhibit germination of *A. thaliana* or *N. tabacum*. The inhibition of microbial growth is sufficient to allow selection of transgenic plants from non‐sterile seeds, avoiding the time‐consuming seed sterilization step and minimizing seed mortality.

We believe that our method provides a useful alternative approach to simplifying *Arabidopsis* transformant screening. It requires minimal labour and can be used with rapid screening methods for BASTA, nourseothricin and hygromycin B resistance. When nourseothricin or hygromycin B are used as the transgene selection reagents, seeds can be sown at high densities and screened on the basis of hypocotyl elongation. For rapid BASTA resistance screening, seeds must be sown at low densities to allow the user to distinguish between green (positive transformant) and pale yellow (wild‐type) cotyledons, and we anticipate that this would also be the case for kanamycin‐based rapid screening (Harrison et al. 2006).

## Author contributions

J.B.Y.H.B. conceived of the concept, designed and executed the experiments, and wrote the manuscript. G.B.G. validated the use of MSTT agar for BASTA‐based screening. M.P. contributed to experimental design and writing the manuscript.

*Acknowledgements –* We thank Dr. Omar Sandoval‐Ibañez (Max Planck Institute of Molecular Plant Physiology, Germany) and Dr. Lars Scharff (University of Copenhagen, Denmark) for their feedback on the manuscript. This project has received funding from the European Union's Horizon 2020 research and innovation programme under Marie Skłodowska Curie Actions Individual Fellowship grant agreement No. 752430 (awarded to JBYHB).

## Supporting information


**Fig. S1.** Terbinafine as an antifungal reagent. Non‐sterile seeds were sown on 0.5× MS agar with added sucrose (1 %, w/v) and different concentrations of terbinafine (indicated). (A) Germination onset is defined as the emergence of cotyledons from the first germinating seeds on each agar plate. (B) Each marker (×) indicates the time at which microbial contamination emerged on individual agar plates. The overall frequency of contamination is summarised at the top of the plot.
**Fig. S2**. Germination of non‐sterile wild‐type seeds on MSTT agar. Non‐sterile seeds of *N. tabacum* and four *A. thaliana* ecotypes were sown on 0.5× MS agar (○) or MSTT agar (□). Plates were observed twice per day and the proportion of germinated seeds was recorded. Germination was defined as cotyledon emergence.
**Fig. S3**. Germination of wild type seeds on 0.5× MS and MSTT agar. Non‐sterile wild‐type seeds for *A. thaliana* ecotypes Columbia (Col 0), Landsberg erecta (Ler 0), Wassilewskija (Ws 0) and Nossen (No 0), and for *N. tabacum* were germinated on either 0.5× MS agar or MSTT agar. Plates were photographed on the seventh day after stratification. A variety of microbial growth is visible on the 0.5× MS agar plates, whereas no microbial growth was observed on MSTT agar.
**Fig. S4**. Adult plants germinated on soil or MSTT agar. Wild‐type *A. thaliana* accessions and *N. tabacum* were germinated on soil or on MSTT agar. Seeds on MSTT agar were stratified directly on the agar plates, while soil germinated seeds were stratified on wet filter paper and then transferred to soil. Seedlings germinated on MSTT agar were transferred to soil 5 days post‐stratification. Adult plants were photographed 20 days post‐stratification, except for Ler‐0 plants which were photographed 14 days post stratification.
**Fig. S5**. Verification of transgene expression with fluorescence imaging. Positive transformants for nuclear encoded chloroplast targeted mCitrine expression (A) and mApple expression (B) were verified with fluorescence imaging.
**Fig. S6**. Verification of Cas9 presence in positive GS2.1/EC transformant lines. Six independent T_1_ plants were identified that showed resistance to hygromycin B. Leaf tissue was sampled for PCR with primers specific to a 1059 bp section of the Cas9 gene. The negative control is tissue from untransformed *A. thaliana* Col‐0 grown on soil. Agarose gel electrophoresis of the PCR products is shown. Gel layout: 1 kb Plus DNA ladder (ThermoFisher Scientific Cat. no. 10787018), T_1_ plants 1‐6, untransformed *A. thaliana* Col 0 (negative control).
**Fig. S7**. Verification of pds3 knockouts. CRISPR‐Cas9‐mediated knockout of the *pds3* gene was performed by stable transfection of *A. thaliana* Col‐0 with the GS2.1/EC construct. The targeted region of *pds3* from three albino *A. thaliana* mutants was sequenced. All three mutants are independent knockout lines. *pds3‐2* is homozygous for a single nucleotide insertion, while *pds3‐1* and *pds3‐3* appear to have heterozygous knockout mutations (i.e. different mutations on each chromosome), indicated by mixed chromatogram peaks after the apparent Cas9 cut site.

## Data Availability

Plasmids created for this study are available at www.addgene.org using the reference numbers described in the text and summarized online in Supporting Information Fig S2. Raw data are available at the following URL: https://www.dropbox.com/s/l9ze4qxcthgq0wo/Behrendorff%20et%20al%20antimicrobials%20raw%20data.zip?dl=0.
