# Supplementary material for: Antimicrobial solid media for screening non‐sterile Arabidopsis thaliana seeds
Source: Physiol Plant. 2020 Mar 14;169(4):586–99. doi: 10.1111/ppl.13079 (PMC7497060; doi:10.1111/ppl.13079)
Supplement: Supplementary file 1 — Fig. S1. Terbinafine as an antifungal reagent. Non‐sterile seeds were sown on 0.5× MS agar with added sucrose (1 %, w/v) and different concentrations of terbinafine (indicated). (A) Germination onset is defined as the emergence of cotyledons from the first germinating seeds on each agar plate. (B) Each marker (×) indicates the time at which microbial contamination emerged on individual agar plates. The overall frequency of contamination is summarised at the top of the plot. Fig. S2. Germination of non‐sterile wild‐type seeds on MSTT agar. Non‐sterile seeds of N. tabacum and four A. thaliana ecotypes were sown on 0.5× MS agar (○) or MSTT agar (□). Plates were observed twice per day and the proportion of germinated seeds was recorded. Germination was defined as cotyledon emergence. Fig. S3. Germination of wild type seeds on 0.5× MS and MSTT agar. Non‐sterile wild‐type seeds for A. thaliana ecotypes Columbia (Col 0), Landsberg erecta (Ler 0), Wassilewskija (Ws 0) and Nossen (No 0), and for N. tabacum were germinated on either 0.5× MS agar or MSTT agar. Plates were photographed on the seventh day after stratification. A variety of microbial growth is visible on the 0.5× MS agar plates, whereas no microbial growth was observed on MSTT agar. Fig. S4. Adult plants germinated on soil or MSTT agar. Wild‐type A. thaliana accessions and N. tabacum were germinated on soil or on MSTT agar. Seeds on MSTT agar were stratified directly on the agar plates, while soil germinated seeds were stratified on wet filter paper and then transferred to soil. Seedlings germinated on MSTT agar were transferred to soil 5 days post‐stratification. Adult plants were photographed 20 days post‐stratification, except for Ler‐0 plants which were photographed 14 days post stratification. Fig. S5. Verification of transgene expression with fluorescence imaging. Positive transformants for nuclear encoded chloroplast targeted mCitrine expression (A) and mApple expression (B) were verified with fluorescence [file PPL-169-586-s001.pdf]

Supporting Figure S1

(A)

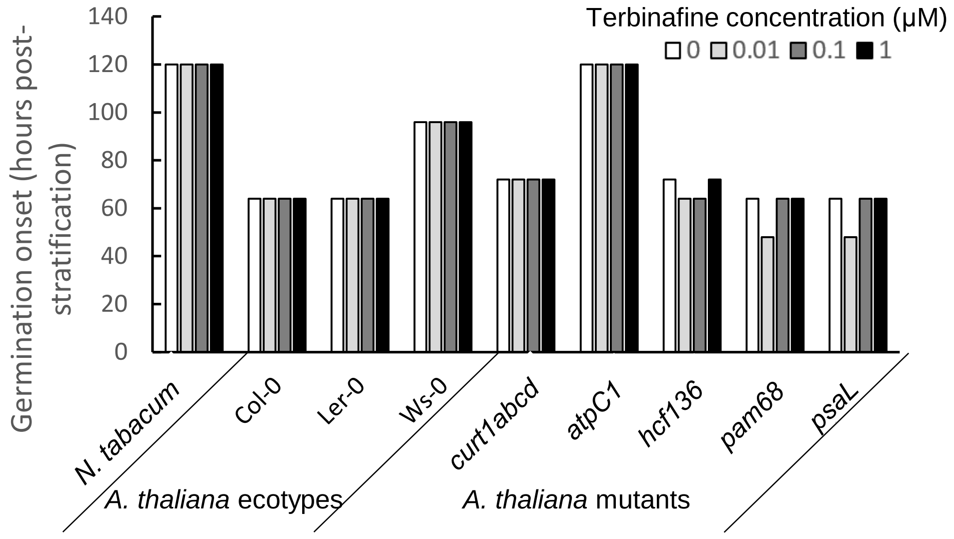

(B)

Terbinafine concentration (µM)	0	0.01	0.1	1
Contamination frequency (plates)	9/9	8/9	6/9	1/9

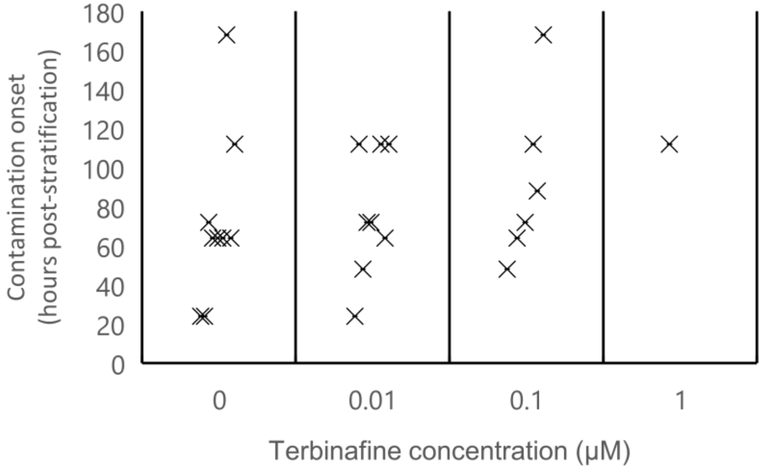

Supporting Figure S1 **Terbinafine as an antifungal reagent.** Non-sterile seeds were sown on 0.5X MS agar with added sucrose (1 %, w/v) and different concentrations of terbinafine (indicated). (A) Germination onset is defined as the emergence of cotyledons from the first germinating seeds on each agar plate. (B) Each marker (×) indicates the time at which microbial contamination emerged on individual agar plates. The overall frequency of contamination is summarised at the top of the plot.

Supporting Figure S2

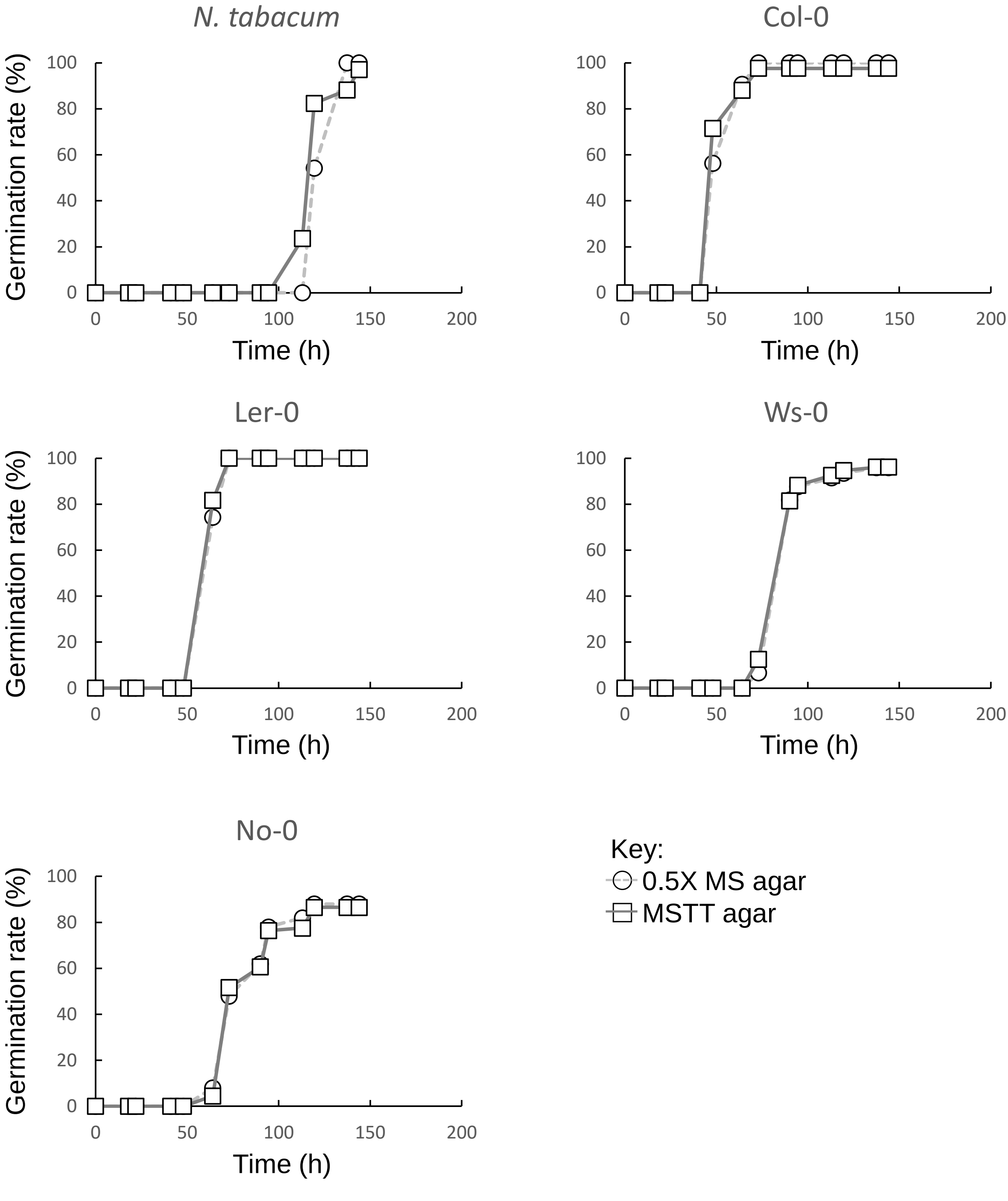

Supporting Figure S2 **Germination of non-sterile wild-type seeds on MSTT agar.** Non-sterile seeds of *N. tabacum* and four *A. thaliana* ecotypes were sown on 0.5X MS agar (○) or MSTT agar (□). Plates were observed twice per day and the proportion of germinated seeds was recorded. Germination was defined as cotyledon emergence.

Supporting Figure S3

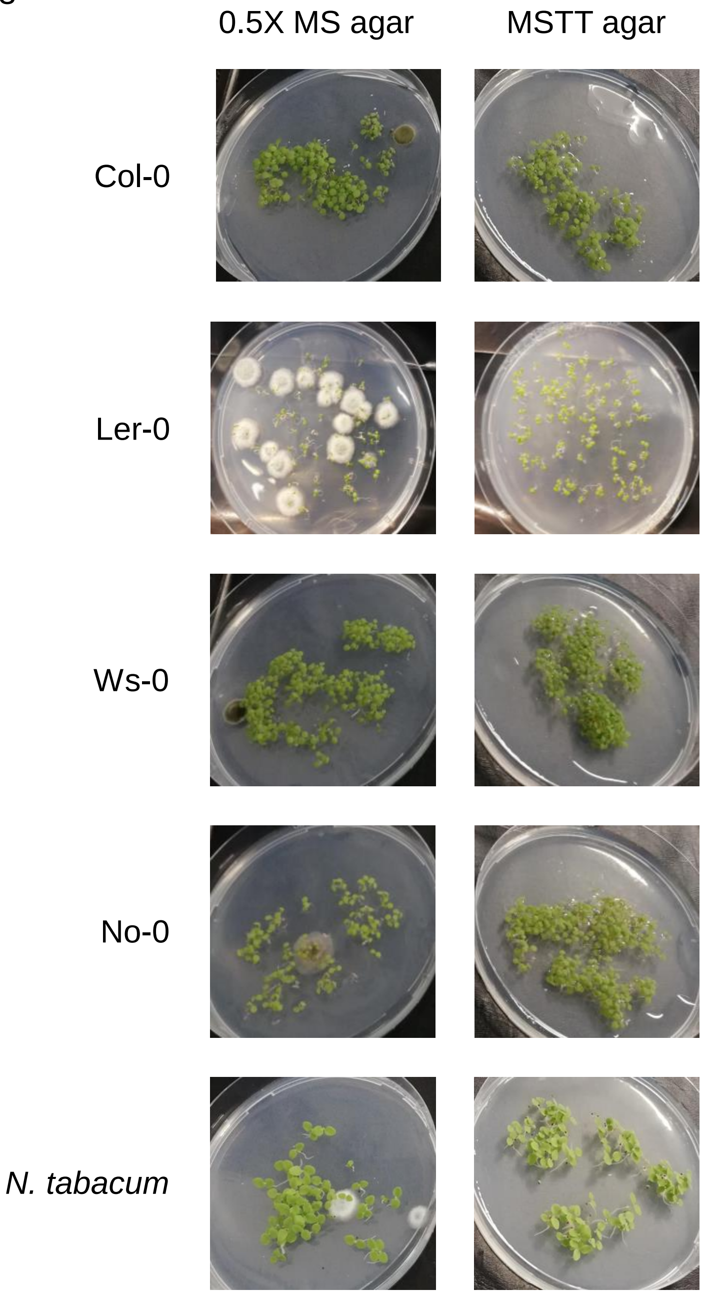

Supporting Figure S3 **Germination of wild-type seeds on 0.5X MS and MSTT agar.** Non-sterile wild-type seeds for *A. thaliana* ecotypes Columbia (Col-0), Landsberg *erecta* (Ler-0), Wassilewskija (Ws-0) and Nossen (No-0), and for *N. tabacum* were germinated on either 0.5x MS agar or MSTT agar. Plates were photographed on the seventh day after stratification. A variety of microbial growth is visible on the 0.5X MS agar plates, whereas no microbial growth was observed on MSTT agar.

Supporting Figure S4

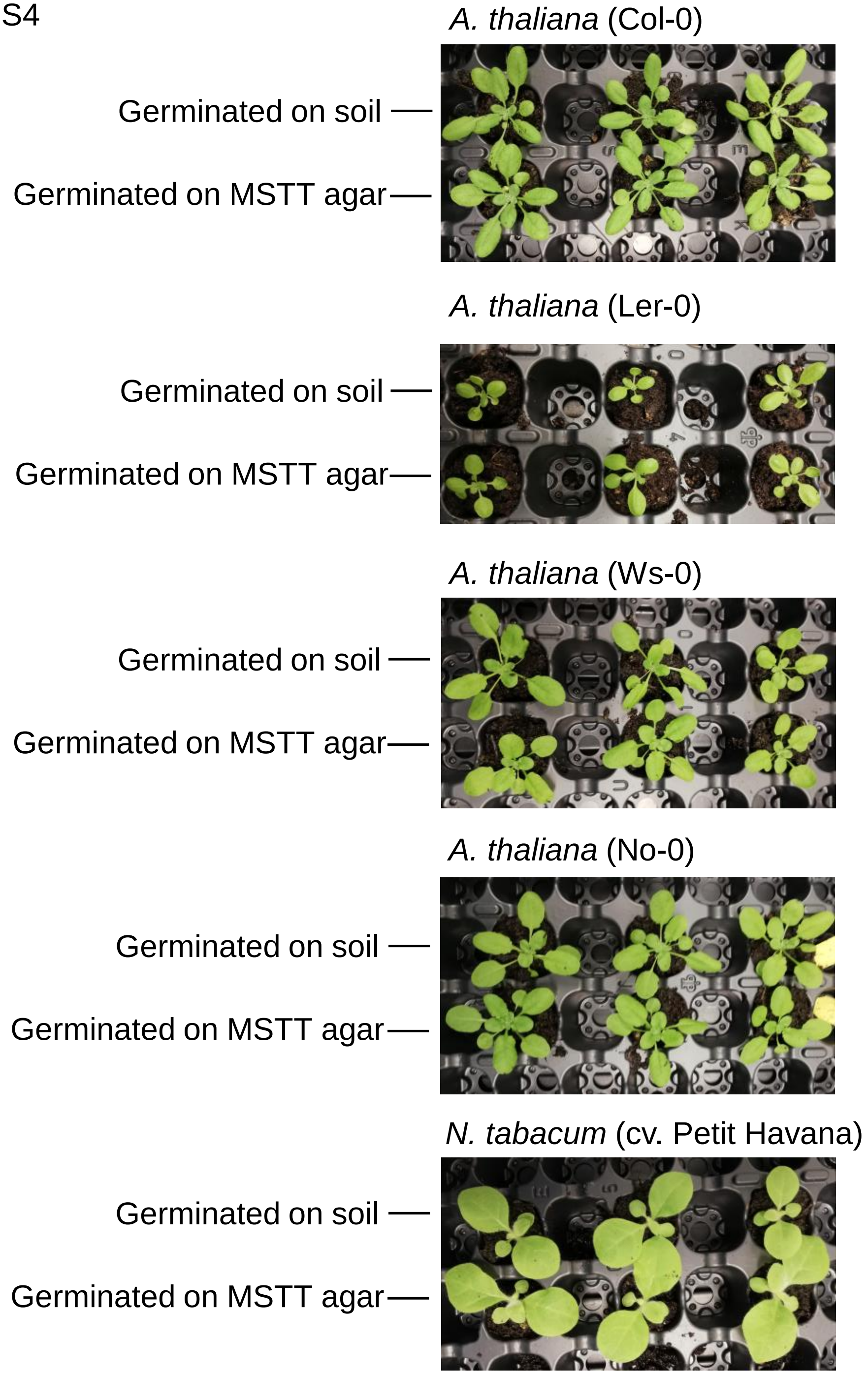

Supporting Figure S4 **Adult plants germinated on soil or MSTT agar.** Wild-type *A. thaliana* accessions and *N. tabacum* were germinated on soil or on MSTT agar. Seeds on MSTT agar were stratified directly on the agar plates, while soil-germinated seeds were stratified on wet filter paper and then transferred to soil. Seedlings germinated on MSTT agar were transferred to soil five days post-stratification. Adult plants were photographed 20 days post-stratification, except for Ler-0 plants which were photographed 14 days post-stratification.

Supporting Figure S5

(A) pN_35S/CTP-mCitrine

White transillumination
No filter

Blue epi illumination
530/28 filter

T_1 seedlings

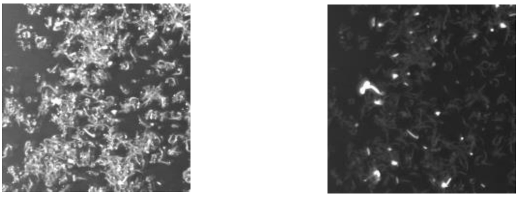

(B) pN_35S/mApple

White transillumination
No filter

Green epi illumination
605/50 filter

T_1 seedlings

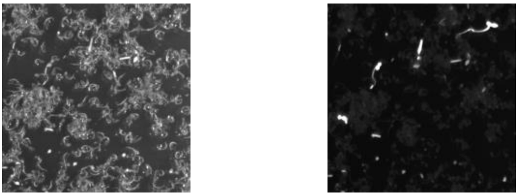

Supporting Figure S5 **Verification of transgene expression with fluorescence imaging.** Positive transformants for nuclear-encoded chloroplast-targeted mCitrine expression (A) and mApple expression (B) were verified with fluorescence imaging.

Supporting Figure S6

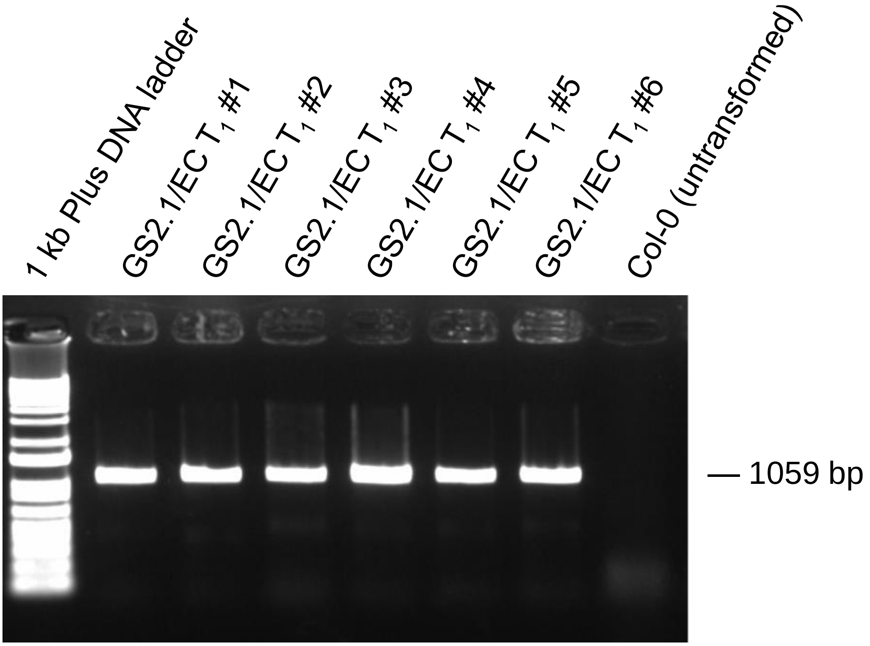

Supporting Figure S6 **Verification of Cas9 presence in positive GS2.1/EC transformant lines.** Six independent T_1 plants were identified that showed resistance to hygromycin B. Leaf tissue was sampled for PCR with primers specific to a 1059 bp section of the Cas9 gene. The negative control is tissue from untransformed *A. thaliana* Col-0 grown on soil. Agarose gel electrophoresis of the PCR products is shown. Gel layout: 1 kb Plus DNA ladder (ThermoFisher Scientific Cat. no. 10787018), T_1 plants 1-6, untransformed *A. thaliana* Col-0 (negative control).

Supporting Figure S7

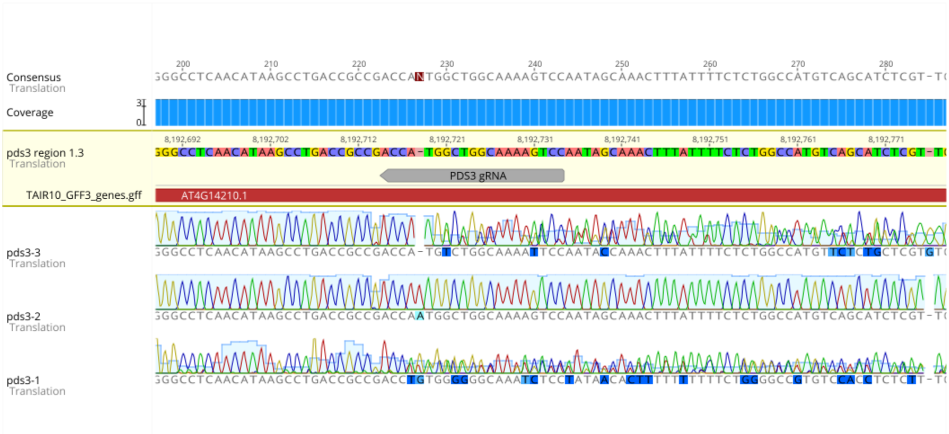

Supporting Figure S7 **Verification of *pds3* knockouts.** CRISPR-Cas9-mediated knockout of the *pds3* gene was performed by stable transfection of *A. thaliana* Col-0 with the GS2.1/EC construct. The targeted region of *pds3* from three albino *A. thaliana* mutants was sequenced. All three mutants are independent knockout lines. *pds3*-2 is homozygous for a single nucleotide insertion, while *pds3*-1 and *pds3*-3 appear to have heterozygous knockout mutations (i.e. different mutations on each chromosome), indicated by mixed chromatogram peaks after the apparent Cas9 cut site.
